# Systematic Review of Pre-Clinical Systems Using Artificial Microenvironments and Anti-Migratory Drugs to Control Migration of Glioblastoma Cells

**DOI:** 10.1017/erm.2024.33

**Published:** 2025-01-23

**Authors:** Hana Selvi, Anke Brüning-Richardson, Davide Danovi

**Affiliations:** 1 Centre for Gene Therapy and Regenerative Medicine, King’s College London, London, United Kingdom; 2 Department of Physical and Life Sciences, School of Applied Sciences, University of Huddersfield Queensgate, Huddersfield, United Kingdom; 3 Department of Basic and Clinical Neuroscience, King’s College London, London, United Kingdom; 4 Migration Biotherapeutics, Cardiff, United Kingdom

**Keywords:** glioblastoma multiforme, nanofibres, hydrogels, anti-migratory drugs, tumour microenvironments

## Abstract

**Background:**

Glioblastoma multiforme (GBM) is the most prevalent primary brain tumour, with an incidence of 2 per 100,000. The standard clinical treatments do not sufficiently target cell migration and invasion, leading to recurrence after surgical resection and resistance after chemotherapy and radiotherapy. Pre-clinical studies are being conducted to construct artificial substrates that can mimic the tumour microenvironment (TME) to prevent GBM cells from migrating along their primary route through blood vessels and white matter tracts. Alongside, targeted therapies using anti-migratory or ‘migrastatic’ drugs are also being developed. This study aimed to review the therapeutic translational strategies emerging from the study of the GBM microenvironment and anti-migratory drugs.

**Methods:**

A systematic literature search was carried out using search key terms and synonyms. Full-paper screening was performed based on specific inclusion and exclusion criteria.

**Results:**

From the systems interrogated, the ‘Nanofibre’ assay is suitable to simulate white matter tracts, while hydrogel-based invasion assays and GBM cerebral organoid (GLICO) mimic the brain extracellular matrix. Inhibitors with anti-migratory activity found in this study are active involving distinct molecular mechanisms and have been tested on cell migration assays.

**Conclusion:**

Overall, we have analysed therapeutic strategies emerging from an artificial GBM TME approach and from the identification of anti-migratory inhibitors. Both carry potential to improve treatment options to prevent tumour dissemination and spread for GBM.

## Graphical abstract

**Figure:**
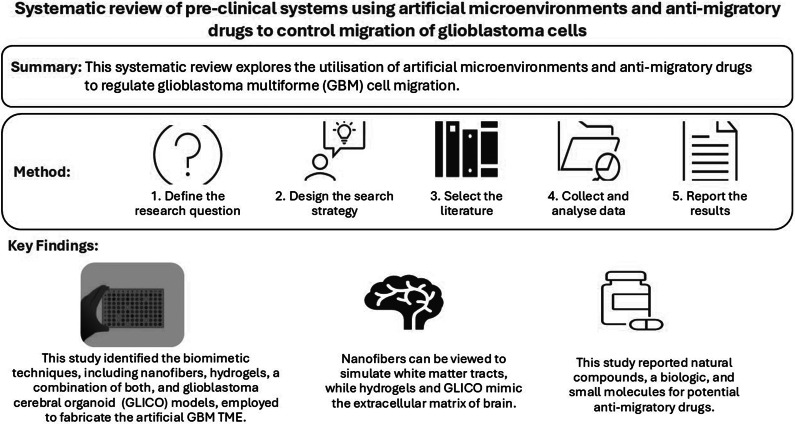

## Introduction

Glioblastoma multiforme (GBM) is the most common and malignant form of primary brain tumour in adults, accounting for about 50% of all gliomas (Refs. 1, 2). GBM is classified as a grade IV glioma, indicating its high level of malignancy and aggressiveness (Ref. 3).

GBM standard therapy combines surgery, radiation therapy, and chemotherapy (Ref. 4). The primary treatment for GBM is surgery, which entails tumour resection without damaging vital brain structures (Refs. 4, 5). Following surgery, radiation therapy aims to kill cancer cells or inhibit their proliferation by delivering high-energy radiation to the tumour site (Refs. 4, 5). It is commonly administered to target remaining highly motile tumour cells. Chemotherapeutic drugs, such as temozolomide (TMZ), are frequently used in combination with radiation therapy. Survival rates for the disease remain extremely poor due to its aggressive nature, recurrence and limited treatment options (Ref. 6). The median overall survival is typically around 12 to 15 months with standard treatment, which usually includes surgery, chemotherapy, and radiation therapy (Ref. 6).

There are ongoing challenges to treat GBM: high infiltration, the exclusivity and immunosuppressive role of the blood–brain barrier (BBB), inter- and intra-tumour heterogeneity (Ref. 5). Surgical resection leaves cells behind that can cause recurrence. Due to their highly infiltrative capability, GBM cells invade into the surrounding normal brain tissue extensively (Ref. 7).

The BBB is a protective barrier that prevents many substances, including chemotherapeutic drugs such as TMZ, from entering the brain and reaching the GBM tumour at sufficient concentrations (Ref. 5); the BBB tight junctions are less than 1 nm, inhibiting the penetration of >98% of small molecules and limiting the effectiveness of systemic therapies (Refs. 5, 8).

Furthermore, GBM has been dubbed a ‘cold’ tumour because it creates an immunosuppressive microenvironment within the tumour, preventing the immune system from recognizing and attacking cancer cells (Ref. 5). Immunosuppressive factors secreted by GBM cells and immune checkpoint molecules contribute to immune evasion and resistance to immunotherapy (Ref. 5).

GBM is characterised by inter and intra-tumour heterogeneity. This means that it exhibits significant genetic and molecular heterogeneity within individual tumours (intratumour heterogeneity) and between different patients’ tumours (intertumoural heterogeneity) (Refs. 5, 9). This heterogeneity promotes varying responses to treatment and the development of resistance (Ref. 5). Since the recurrence of tumours ultimately leads to the death of the patient and current treatment approaches are inadequate to control tumour spread,

Novel GBM treatment strategies are currently being developed to control GBM migration and invasion using artificial GBM tumour microenvironments (TMEs) and anti-migratory drugs (Refs. 10, 11). This review identifies and evaluates the types of biomimetic technologies currently under pre-clinical research; we also assess pre-clinical studies on anti-migratory therapeutics, the effect of which was observed in vitro using migration assays. The goal of this work is thus to thoroughly report previously published studies in this space.

## Methods

Preferred Reporting Items for Systematic Reviews and Meta Analysis (PRISMA) guidelines were used for this study (Ref. 12). A literature search was conducted using the *PubMed, Medline, and Web of Science* database up to June 2023, with results restricted to articles written in the English language. The original search terms included GBM, glioma, glioblastoma, glioblastoma cells, brain tumour cells, brain tumour, TMEs, extracellular matrix (ECM), nanofibres, cell trap, migration, migrating, migrate, anti-migration effect, anti-migration, anti-migratory and inhibit. Studies were selected for inclusion in the systematic review and analysis if: (a) the literature was available full-text in English; (b) articles were published in the last decade from January 2013 to May 2023; (c) there was an impact of interventions (systems using artificial microenvironments or anti-migratory drugs) capable of controlling or inhibiting the migration of GBM; (d) there was a measurement outcome observed from the migration assay of GBM in the research articles; (e) migration assays were carried out *in vitro* or *in vivo* for anti-migratory drugs.

**Figure 1.**
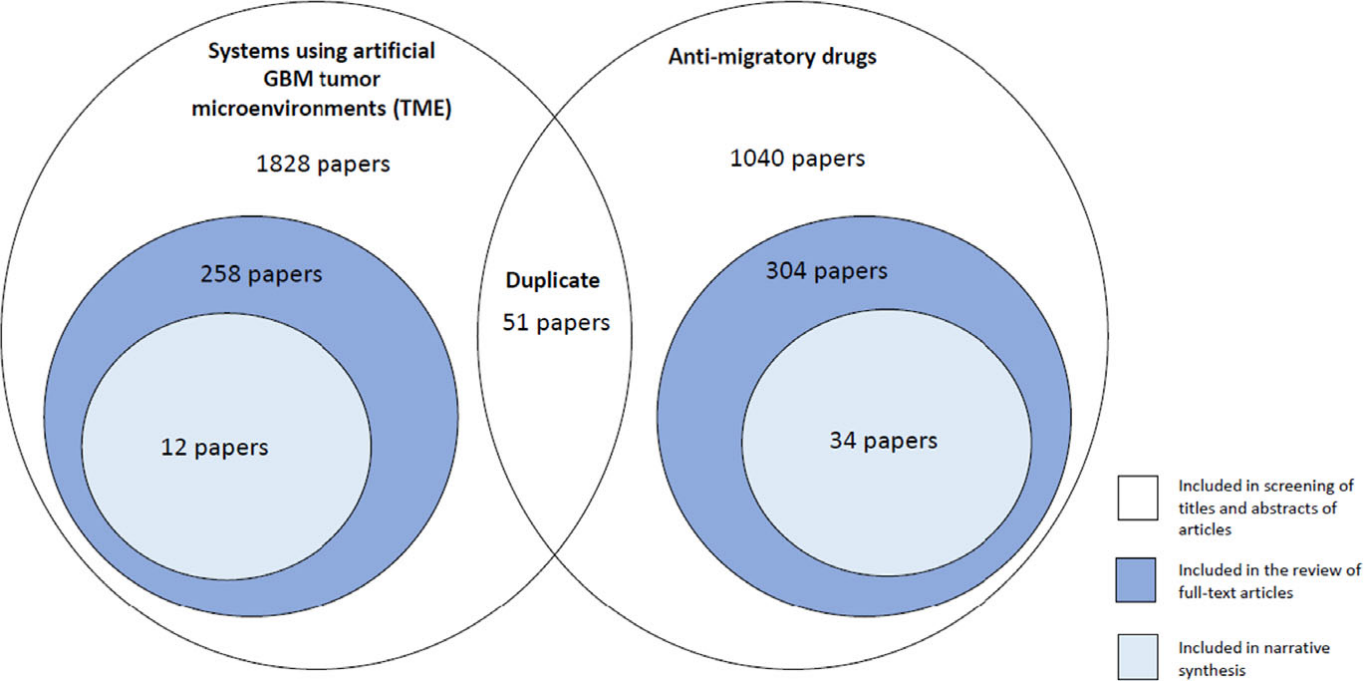
*Venn* diagram for selection and number of studies for systems using artificial glioblastoma multiforme tumour microenvironment (GBM TME) and anti-migratory drugs (therapeutic agents that specifically target and inhibit GBM cells to migrate and invade surrounding healthy brain tissue).

**Figure 2.**
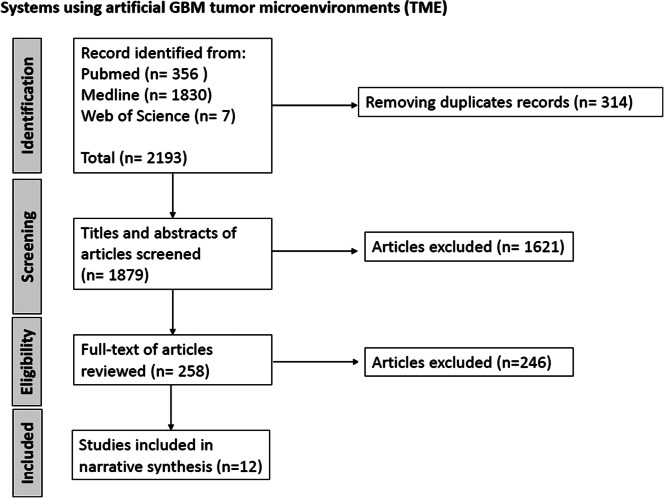
Flow chart for selection study process and literature review of the system using artificial tumour microenvironment (TME).

**Figure 3.**
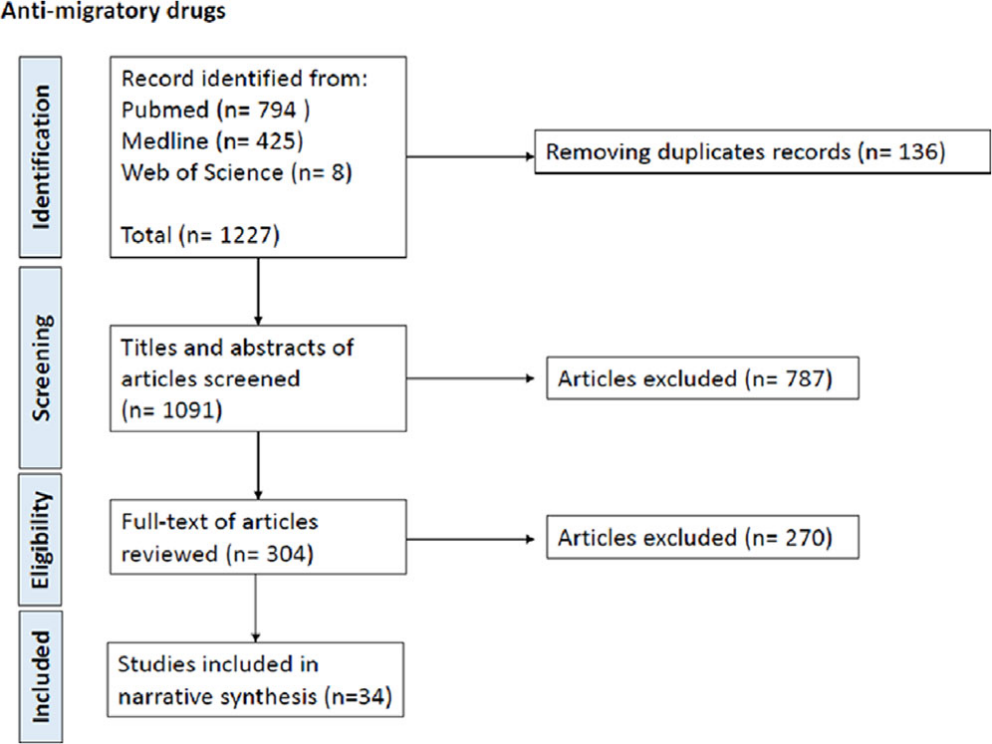
Flow chart for selection study process and literature review of anti-migratory drugs.

The PubMed, Medline, and Web of Science search yielded a total of 1879 articles for systems using artificial TME and 1091 articles for anti-migratory drugs and proceeded in the first stage to screen and select studies based on titles and abstracts. After the initial screening, 258 papers of systems using artificial GBM TME and 304 papers of anti-migratory drugs were selected to enter the full paper screening based on the inclusion and exclusion criteria (see Figure 1). From the extracted data, 12 papers of systems using artificial GBM TME and 34 papers of anti-migratory drugs were included in the data analysis stage using narrative synthesis. Titles for all selected papers are available in Appendix Tables S1 and S2. The complete PRISMA flow charts are presented in Figure 2 and Figure 3 (Refs. 12, 13).

Assessments for the quality of *in vitro* and *in vivo* studies were carried out separately according to the method. Results of the assessments for the quality of the studies are available in Appendix Tables S3 and S4. All studies included in this review had passed our criteria for quality based on the assessment with the highest score and meeting the criteria in Appendix Tables S3 and S4.

## Discussion

### Biomimetic techniques used in systems exploring artificial microenvironments for GBM treatment

#### Using nanofibres to mimic white matter tracts

**Figure 4.**
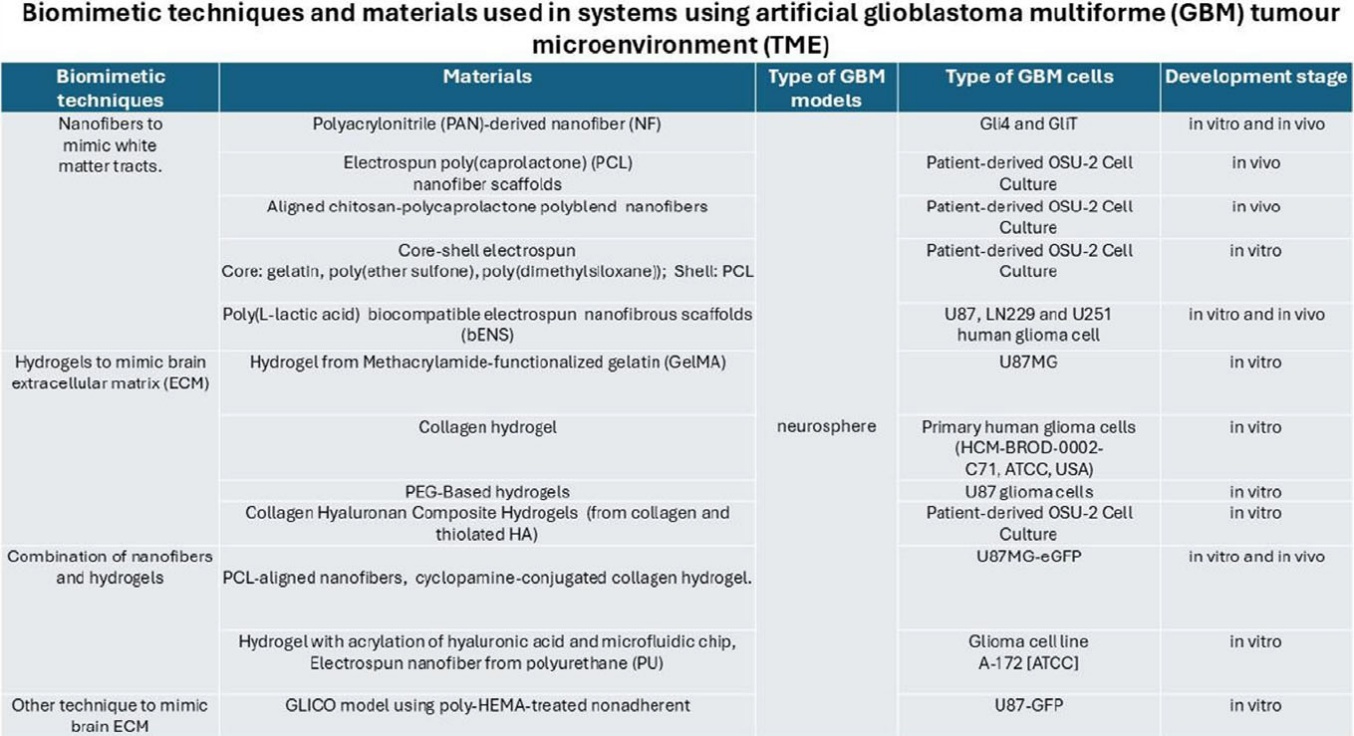
Summary of biomimetic techniques and materials used in systems using artificial glioblastoma multiforme (GBM) tumour microenvironment (TME).

Nanofibres, as a biomimetic technique, are designed to mimic white matter tracts as one of the main pathways of GBM migration (Refs. 14–18). White matter tracts, as extracellular factors for GBM migration and invasion, have a unique topography to promote GBM migration (Ref. 19). The summary of materials and types of GBM models using nanofibres to mimic white matter tracts, along with their development stages, is available in Figure 4.

The choice of nanofibres as a material for scaffolds appears to be promising due to the possibility to fit the architecture for the GBM cells invading the ECM in a nanosized with a high-aspect ratio of fibres (Refs. 16, 20). These high-aspect ratio fibres, which were derived from a chitosan and polycaprolactone (PCL) blend, might be able to promote glioblastoma cell migration. These fibres’ alignment mimicked the ECM environment naturally by giving the cells a physical cue to move along. This alignment promoted directional migration of the cells and may have implications for understanding the behavior of glioblastoma cells in their native tissue microenvironment (Ref. 16). In the researcher literature, PCL was commonly used as the material for biomimetic techniques using nanofibres. The choice of PCL as a ‘building block’ for nanofibre scaffolds is justified by its high biocompatibility, good mechanical strength, and slow biodegradability as a synthetic polymer (Refs. 21, 22). Because PCL is highly biocompatible, it is unlikely to have harmful effects or be toxic when it comes into contact with biological systems. Good mechanical strength is possessed by PCL, which is crucial for the stability and structural integrity of the scaffolds utilized in this investigation. Scaffolds made of PCL are expected to retain their structural integrity for a considerable amount of time due to their slow biodegradability (Unal et al., 2020).

Rao et al. (2013) showed the superiority of PCL nanofibre in affecting GBM cell migration speed compared to other materials with a higher impact on the rate of migration (Ref. 17). This work addressed the use of core-shell electrospun nanofibres composed of different materials to mimic the topography or physical makeup of the brain’s white matter tracts and investigate how it affects the migration patterns of cancerous brain tumours, specifically concentrating on GBM cells (Ref. 17).

Furthermore, other reported nanofibre materials such as polyacrylonitrile (PAN), poly(L-lactic acid), gelatin, poly(ethersulfone), and poly(dimethylsiloxane) can be alternatives for mimicking the topography of white matter tracts and affecting the GBM migration. Materials based on PAN, poly(L-lactic acid), gelatin, poly(ethersulfone), and poly(dimethylsiloxane) had each unique properties, such as mechanical strength, surface roughness, biocompatibility, and degradation rate. In the context of this study, PCL nanofibres might have exhibited properties that appeared more similar to the natural white matter tracts in the brain, allowing for better mimicry of the topography (Ref. 17).

The review of five identified research papers using nanofibres (see Appendix Table S5) indicates there are multiple ways in which nanofibres can imitate white matter tracts and act as scaffolds for GBM cell migration. These include topographical similarity to the brain’s natural white matter tracts and the ability of similar-looking nanofibres’ mechanical properties to create an environment that mimics white matter tracts. The majority (4 out of 5) had been tested *in vivo* in xenograft GBM cell model in rodents and prevented migration of GBM cells through the white matter tract *in vivo.* To accomplish this in vivo procedure, human GBM cells could be implanted into the brains of rodents to create a xenograft GBM model. GBM cell migration could then be tracked along the nanofibres to determine how well the nanofibres block or divert GBM cell migration through the white matter tracts. The utilisation of nanofibres was facilitated by several mechanisms: (1) topographical resemblance to the brain’s native white matter tracts; (2) mechanical properties of nanofibres with comparable attributes that can generate a microenvironment that resembles white matter tracts; and (3) guidance cues: nanofibres can be functionalized with a range of bioactive molecules or signalling cues that can direct GBM cell migration; nanofibres can mimic white matter tracts and function as migration scaffolds for the GBM cells. No first-in-human experiment has been described to our knowledge.

#### Using hydrogels to mimic ECM around blood vessels

Blood vessels are one of the main routes for GBM cell migration in addition to white matter tracts. Figure 4 provides a summary of the materials and types of GBM models that utilise hydrogels to replicate the brain ECM, along with their stages of development. The use of hydrogels is a biomimetic technique designed to mimic the ECM, which is important in regulating GBM cell migration. Collagen as a material for producing hydrogels is a biologically relevant choice (Ref. 23). In addition, collagen also plays a role in the GBM tissue’s mechanical strength, causing the invasion of GBM cells, due to the contribution of collagen to the structural integrity and mechanical properties of glioblastoma (GBM) tissue. Collagen is a major component of the ECM and provides structural support to tissues (Ref. 23). One study used gelatin methacrylamide (GelMA) as a hydrogel because of its ability to encapsulate molecules (e.g., therapeutic drugs, growth factors, or other signaling molecules) and its suitable permeability (Ref. 24). GelMA hydrogel can control the release of drugs, growth factors or encapsulate cells (e.g., tumour cells, stem cells, or other cell types relevant to brain tumour culture and therapy), making them preferred materials for bioengineering (Ref. 24). The use of polyethylene glycol (PEG) as a component for producing hydrogels can also be an alternative because PEG has been approved for human use by the FDA (Ref. 25). According to Sahan et al., 2022, the use of polyethylene glycol (PEG) as a hydrogel material was relevant to mimic the mechanical properties of the native TME, such as stiffness and viscoelasticity. In addition, PEG has good stiffness tunability for mechanics studies (Ref. 26).

In Ngo et al., hydrogels were used as perivascular niches (PVN) models in GBM aimed to recreate the microenvironment surrounding blood vessels in the brain, where GBM cells often migrate and invade into (Ref. 27). PVNs are specialized regions around blood vessels that provide a supportive niche for GBM cells, promoting their survival, migration, and resistance to therapy (Ref. 27). To construct PVN models, hydrogels are engineered to mimic the composition and physical properties of the brain ECM (Ref. 27). The spread of GBM cells due to GBM cell migration causes co-option or blood vessel damage. Diversion with newly formed vasculature from hydrogel will prevent co-option due to migration of the GBM cells to the PVN (Ref. 27).

Interestingly, in Yao et al.’s paper, the use of hydrogels as a biomimetic technique is uniquely combined with using an electric field which influenced the direction and migration velocity of GBM cells (Ref. 28). Using this electrical field helped to change the random order into a clear anodal migration of GBM cells and increased the migration velocity proportional to the increase of the electrical field (Ref. 28). This result is in accordance with the literature regarding migratory cues that cause GBM migration to vary, where electrical cues can be considered extracellular factors (Ref. 29).

Wang et al. also reported on the influence of hydrogel stiffness on GBM cell migration. The study showed that hydrogel stiffness affected the migration of GBM cells due to the expression of matrix metalloproteinase-9 (MMP-9) (Ref. 25). According to the study, glioblastoma cell migration was significantly impacted by the stiffness of the hydrogel. In comparison to softer hydrogels, stiffer hydrogels caused glioblastoma cells to migrate more. This finding suggested that the mechanical properties of the TME had a major impact on the behaviour of glioblastoma cells, particularly on migration (Ref. 25).

All the studies reviewed above were at the pre-clinical *invitro* stage (see Appendix Table S6); experiments need to be confirmed in *vivo* to assess the clinical relevance of the initial findings.

#### Using a combination of nanofibre and hydrogels to mimic the brain ECM

Figure 4 summarises the materials and types of GBM models that utilise a combination of nanofibres and hydrogels to replicate the brain’s ECM, along with their stages of development. The nanofibres in Jain *et al* and Lee *et al’s* experiments aimed to mimic the white matter tract and encourage tumour cells into moving towards extracortical locations and away from the primary tumour site (Refs. 30, 31). In Jain et al.’s study, the nanofibres conduits contained two components, namely PCL/polyurethane guidance conduit and cyclopamine-conjugated collagen hydrogel which acted as an apoptotic ‘tumour sink’ in the extracortical space (Ref. 30). The term “tumour sink” describes the deliberate rerouting of tumour cells (GBM cells) towards a particular location or substance that has the ability to efficiently trap or eradicate them (Ref. 30). Sonic hedgehog (Shh) pathway antagonist Cyclopamine was chosen as a cell trap, and cyclopamine-conjugated collagen hydrogel was implanted above the fibre film of tumour guidance conduits (Ref. 30). In the study by Lee et al., there was an addition of the use of a polydimethylsiloxane (PDMS)-based microfluidic chip to monitor the migration of GBM cells (Ref. 31). In this study, the small-scale device or platform designed to manipulate and control the flow of fluids at a microscale level was integrated with a gel-like material that mimics the properties and structure of natural tissues (a biomimetic hydrogel). The integration of the microfluidic chip with the biomimetic hydrogel allowed for precise control and monitoring of cell alignment and migration in a three-dimensional (3D) environment (Ref. 31).

In this experiment, an *in vivo* trial was performed by inserting nanofibre conduit or scaffold into a rat brain with GBM. In this paper, a trial was conducted in a living organism (in vivo) by inserting a nanofibre conduit or scaffold into the brain of a rat with GBM. This experiment’s purpose was likely to investigate the nanofibre conduit or scaffold’s potential in guiding and treating brain tumour cells. This experiment showed that 30 μM cyclopamine was only toxic to GBM cells. Data from the NMR experiment revealed that cyclopamine did not diffuse into the surrounding healthy brain tissue and was only confined in the hydrogel, harming GBM cells that migrated into it (Ref. 30).

Lee et al. also used an additional material by adding microfluidic chips. HA hydrogel was inserted in a microfluidic chip with various flow conditions to mimic a cellular microenvironment. Besides that, the system used in this experiment was easy to modify, the hydrogel composition in a microfluidic system was modified by employing RGD (arginine (R), glycine (G), and aspartic acid (D)) peptides to promote cell adhesion along with MMP-sensitive peptides for monitoring matrix remodeling (Ref. 31). This study observed the effect of growth factor addition, for instance, vascular endothelial growth factor (VEGF)-loaded flow into the cell. Based on the experiment, adding growing factors such as VEGF in the microfluidic chips may represent one driving force of cell migration. This information may help understand the migration of GBM cells and metastasis of cancerous tissue (Ref. 31).

Overall, developing the combination of these biomimetic techniques is promising as it can provide a biomimetic environment that closely mimics the native brain tissue by the ability of the nanofibres to provide fibrous architecture and physical cues for migration and the ability of hydrogels to offer a more complex three-dimensional environment (see Appendix Table S7). In addition, the availability of materials added to increase the function of this biomimetic combination also enhances the prospects of this biomimetic technique.

### Anti-migratory drugs for GBM treatment

Most potential anti-migratory drugs identified based on our criteria (20 out of 33) are derived from natural compounds. Based on data over the last three decades, nearly 80% of FDA-approved drugs for cancer treatment are either natural products or derivatives, and natural compounds are prospective and promising for the development of migrastatic drugs (Ref. 32).

**Figure 5.**
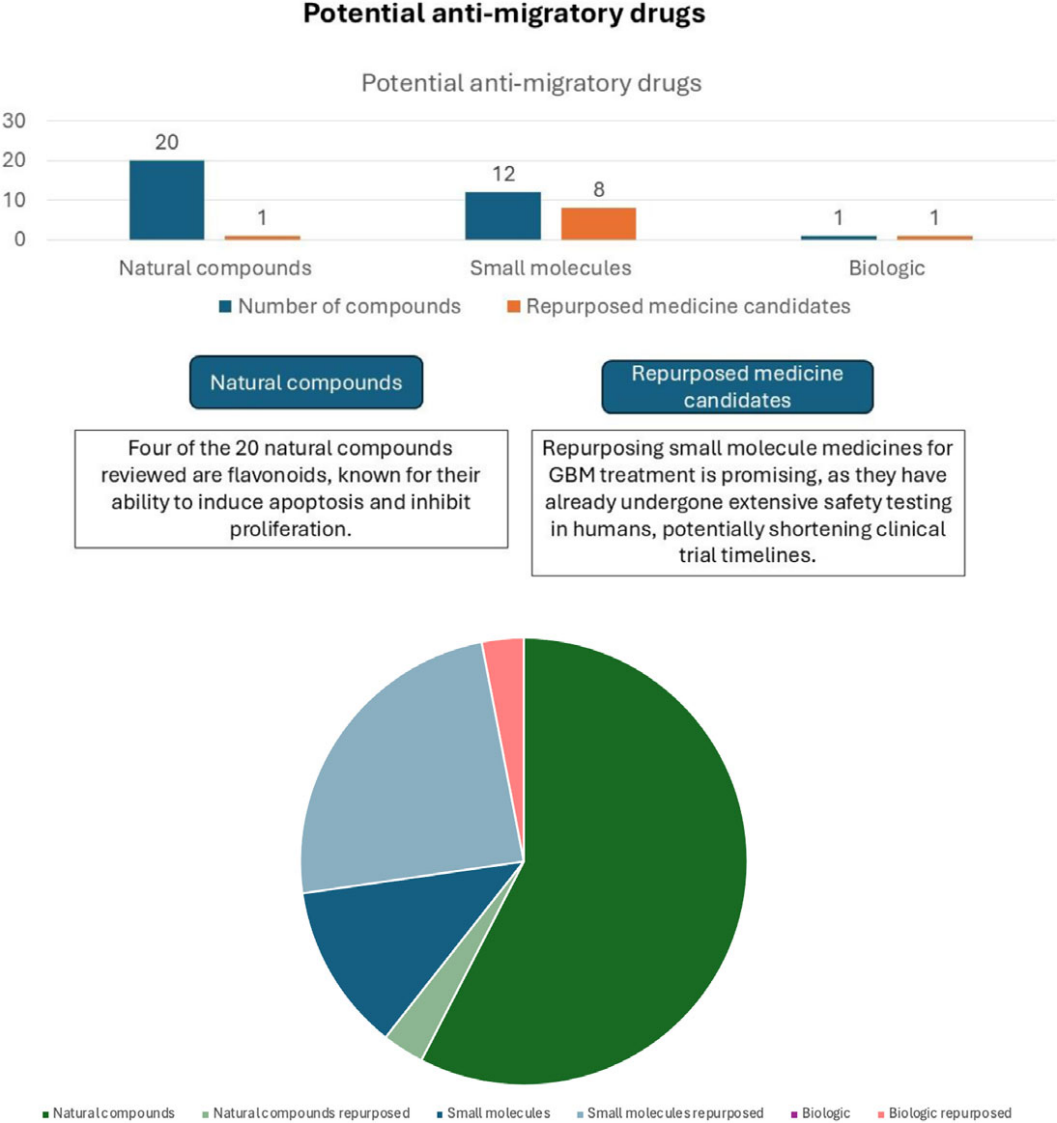
Bar chart of potential anti-migratory drugs and candidates for repurposed medicines.

Four out of the 20 natural compounds from selected papers are flavonoids. Based on the literature, flavonoids are important in cancer treatment because they can induce apoptosis and inhibit proliferation (Ref. 33). In Li et al. ‘s 2022, “flavonoids” refer to a group of natural compounds found in various fruits, vegetables, and plants. Flavonoids are known for their diverse biological activities and have been extensively studied for their potential health benefits, including their anticancer properties. The studies from selected papers also showed the ability of carvacrol, naringin, a combination of rutin and quercetin, and a combination of luteolin and apigenin to inhibit GBM migration and proliferation, also able to induce apoptosis of GBM cells (Refs. 34–38). Some of the small molecules (8 out of 12) were potential repurposed medicines for GBM treatment, and there was also one literature that used repaglinide which was a biologic (see Appendix Table S9). Overall, the potential anti-migratory drugs have various sources of compounds such as from natural compounds, small molecules, and biologics, with the most trend coming from natural compounds. Figure 5 presents a bar chart illustrating potential anti-migratory drugs and candidates for repurposed medicines.

Most of the potential anti-migratory compounds have been studied in detail to understand the molecular mechanism causing inhibition of GBM cell migration and invasion (see Appendix Table S8 and S9). GBM migration is characterized by many molecular mechanisms reflective of the complexity and heterogeneity of the disease (Ref. 29). GBM cells can exploit the ECM to facilitate migration, and they can secrete proteolytic enzymes, such as matrix metalloproteinases (MMPs), which degrade ECM components and create paths for cell migration (Refs. 11, 35, 39–41). They can also switch from a mesenchymal-like to amoeboid-like way of cell migration when challenged with specific inhibitors. Moreover, several compounds were targeting VEGF and epidermal growth factor receptor (EGFR), as well as various signaling pathways to inhibit GBM cell migration (Refs. 35, 42–44). Studying the molecular mechanism behind the inhibition of GBM cell migration is crucial to develop effective anti-migratory drugs. By understanding the molecular mechanisms, we can design targeted therapy based on molecular mechanisms that are effective in blocking process mechanisms, increasing efficacy, and supporting the development of targeted therapy for GBM.

The ability of CNS drugs to cross the BBB is one of the challenges in the current treatment (Refs. 45, 46). Two studies, namely alantolactone (ATL) and curcumin, performed assays to determine these two compounds’ ability to penetrate the BBB (Refs. 46, 47). The ability of ATL to penetrate the BBB was determined by LC–MS/MS assay using ATL and the collected cerebrospinal fluid. Based on the experimental results, it showed ATL was able to penetrate the BBB. Meanwhile, the BBB study for the curcumin by Razali et al. was carried out using the Absorption, Distribution, Metabolism, Excretion, and Toxicity (ADMET) prediction from two platforms, namely AlzPlatform (www.cbligand.org/AD/) and ADMETlab 2.0 (https://admetmesh.scbdd.com/). The ADMET predicted the ability of a compound to penetrate the BBB based on its pharmacokinetic properties and toxicity (ADMET) (Ref. 47). In this experiment, curcuminoid analogs FLDP-5 and FLDP-8 were used, and it showed that these two curcuminoid analogs were predicted to be BBB permeable (Ref. 47). Since it is essential to identify the ability of anti-migratory compounds to penetrate the BBB to increase the efficacy of GBM treatment, future studies should test the ability of compounds to penetrate the BBB.

### Implications for future GBM treatment

**Figure 6.**
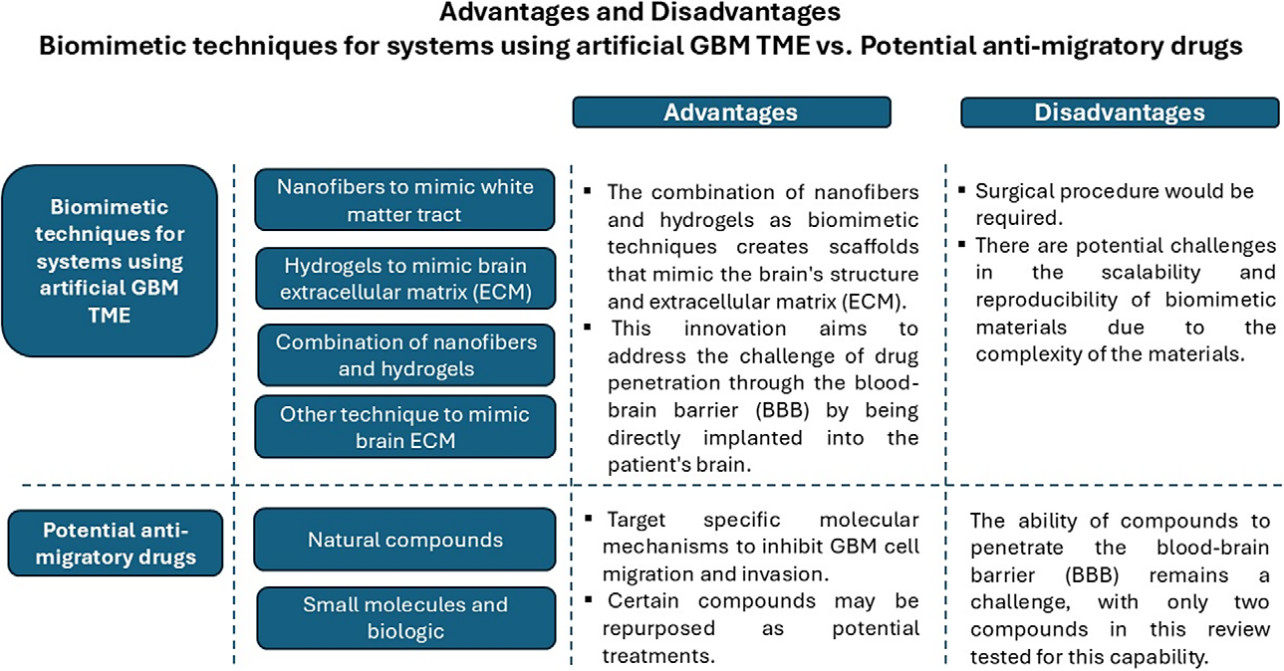
Advantages and disadvantages of biomimetic techniques for systems using artificial GBM TME versus potential anti-migratory drugs.

Figure 6 illustrates the advantages and disadvantages of biomimetic techniques for systems using artificial GBM TME compared to the use of potential anti-migratory drugs. The combination of using nanofibre and hydrogels as biomimetic techniques allows an accurate representation of the structure and ECM of the brain (Refs. 30, 31). These two biomimetic techniques can be developed into a single medical device implanted into the patient’s brain to divert GBM migration through this artificial TME. A surgical procedure would be required to implant a single medical device into the patient’s brain to redirect GBM migration through an artificial TME, such as nanofibres. The feasibility of implanting a single medical device into the brain to divert GBM migration through an artificial TME is still an active area of research. Bringing this concept to reality will require additional study and advancements in the domains of materials science, surgery, and GBM biology. Apart from using nanofibres and hydrogels, adding cyclopamine to hydrogels can be used as a cell trap that kills GBM cells, which is valuable (Ref. 30). The study’s use of cyclopamine in hydrogels will be valuable since it enables targeted cell trapping, increased cytotoxicity, and regulated drug delivery to GBM cells. This strategy minimised harm to healthy brain tissue while specifically targeting and eliminating tumour cells, potentially providing therapeutic benefits for the treatment of GBM. This innovation may be able to overcome drug delivery to the brain because this technique is directly implanted into the patient’s brain. However, it is essential to note that the development and implementation of such medical devices require further extensive research, testing, and regulatory approval to ensure their safety and efficacy.

Anti-migratory drugs are extremely promising candidates for the improved treatment of GBM because this additional treatment specifically targets cell migration and invasion, which is not included in the current standard therapy and prevents tumour dissemination and recurrence (Ref. 11). By a complementary approach of combined anti-migratory and cytotoxic targeting in GBM management, anti-migratory drugs, especially those predicted to cross the BBB such as alantolactone and curcuminoid analogs, may be a promising tool for targeted GBM therapy (Refs. 46, 47). As a variety of compounds have already gone through clinical trials or are known as repurposed drugs for previously approved indications (see Appendix Table S9), this can potentially shorten clinical trials and speed up translation to the clinic; repurposed anti-migratory drugs for which mode of action are already known are therefore of specific interest for application in the treatment of GBM (Refs. 39, 48–50). However, further research and clinical trials are needed to fully evaluate the safety and efficacy of these drugs and their potential in combination with other treatment modalities.

## Conclusion

Systems using artificial GBM TME may be explored to control the migration of GBM by diverting GBM migration through a new artificial GBM TME. The new artificial GBM TME must be able to mimic the brain ECM to attract migratory activity to the artificial GBM TME. It is our view that an optimum approach to incorporate targeting cell migration and invasion in GBM management is a combination of systems using artificial GBM TME based on nanofibres and hydrogels.

Furthermore, anti-migratory drugs represent excellent candidates for GBM treatment by specifically inhibiting migration and invasion of GBM cells. This ability has been confirmed by a variety of 2D and 3D migration assays, and for most compounds, the molecular mechanisms underlying drug activity have been identified. In order to determine the potential of anti-migratory medications to cross the BBB and reach the tumour site, it is essential to assess their ability to do so in a clinical setting. Systems using artificial GBM TME and anti-migratory drugs can be potential candidates for GBM treatment and as such help addressing a substantial unmet need to improve the survival and quality of life of GBM patients.

## Supporting information

Selvi et al. supplementary material Selvi et al. supplementary material

## Supplementary material

For supplementary material accompanying this paper visit http://doi.org/10.1017/erm.2024.33. click here to view supplementary material
