# Supplementary material for: Systematic Review of Pre-Clinical Systems Using Artificial Microenvironments and Anti-Migratory Drugs to Control Migration of Glioblastoma Cells
Source: Expert Rev Mol Med. 2025 Jan 23;27:e6. doi: 10.1017/erm.2024.33 (PMC11803519; doi:10.1017/erm.2024.33)
Supplement: Selvi et al. supplementary material [file S1462399424000334sup001.docx]

**TABLES**

**Appendix Table S1** Selected papers for systems using artificial GBM TME

| **Titles** | **References** |  |
| --- | --- | --- |
|  |  |  |
|  |  |  |
| A novel 3D nanofibre scaffold conserves the plasticity of glioblastoma stem cell invasion by regulating galectin-3 and integrin-β1 expression. | (52) |  |
| Glioma-astrocyte interactions on white matter tract-mimetic aligned electrospun nanofibers. | (15) |  |
| Aligned Chitosan-Polycaprolactone Polyblend Nanofibers Promote the Migration of Glioblastoma Cells. | (16) |  |
| Mimicking white matter tract topography using core–shell electrospun nanofibers to examine migration of malignant brain tumors | (17) |  |
| Electrospun nanofibrous scaffolds increase the efficacy of stem cell-mediated therapy of surgically resected glioblastoma. | (14) |  |
| Guiding intracortical brain tumour cells to an extracortical cytotoxic hydrogel using aligned polymeric nanofibers. | (31) |  |
| Integration of microfluidic chip with biomimetic hydrogel for 3D controlling and monitoring of cell alignment and migration: Biomimetic Hydrogel. | (32) |  |
| Multidimensional hydrogel models reveal endothelial network angiocrine signals increase glioblastoma cell number, invasion, and temozolomide resistance. | (28) |  |
| Collagen Matrices Mediate Glioma Cell Migration Induced by an Electrical Signal. | (29) |  |
| Bioengineered 3D Brain Tumor Model To Elucidate the Effects of Matrix Stiffness on Glioblastoma Cell Behavior Using PEG-Based Hydrogels. | (26) |  |
| Glioblastoma Behaviors in Three-Dimensional Collagen-Hyaluronan Composite Hydrogels. | (23) |  |
| Glioblastoma and cerebral organoids: development and analysis of an in vitro model for glioblastoma migration. | (53) |  |

**Appendix Table S2** Selected papers for anti-migratory drugs

| **Titles** | **References** |
| --- | --- |
| The flavonoid rutin and its aglycone quercetin modulate the microglia inflammatory profile improving antiglioma activity. | (39) |
| Differential Effects of Wedelia chinensis on Human Glioblastoma Multiforme Cells. | (37) |
| Inhibition of TRPM7 by carvacrol suppresses glioblastoma cell proliferation, migration, and invasion. | (54) |
| Natural dietary compound naringin inhibits glioblastoma cancer neoangiogenesis. | (35) |
| Naringin suppresses cell metastasis and the expression of matrix metalloproteinases (MMP-2 and MMP-9) via the inhibition of ERK-P38-JNK signaling pathway in human glioblastoma. | (36) |
| Berberine inhibits glioma cell migration and invasion by suppressing TGF-beta1/COL11A1 pathway. | (55) |
| Kukoamine A inhibits human glioblastoma cell growth and migration through apoptosis induction and epithelial-mesenchymal transition attenuation. | (56) |
| Ginsenoside Rh2 inhibits metastasis of glioblastoma multiforme through Akt-regulated MMP13. | (41) |
| Verbascoside suppresses the migration and invasion of human glioblastoma cells via targeting c-Met-mediated epithelial-mesenchymal transition. | (57) |
| Curcumin piperidone derivatives induce anti-proliferative and anti-migratory effects in LN-18 human glioblastoma cells. | (48) |
| Curcumin suppresses glioblastoma cell proliferation by p-AKT/mTOR pathway and increases the PTEN expression. | (58) |
| Anti-Migratory Effect of Vinflunine in Endothelial and Glioblastoma Cells Is Associated with Changes in EB1 C-Terminal Detyrosinated/Tyrosinated Status. | (59) |
| Tubeimoside-1 Inhibits Glioblastoma Growth, Migration, and Invasion via Inducing Ubiquitylation of MET. | (60) |
| Alantolactone, a natural sesquiterpene lactone, has potent antitumor activity against glioblastoma by targeting IKKβ kinase activity and interrupting NF-κB/COX-2-mediated signaling cascades. | (47) |
| Cannabisin D from Sinomenium Acutum Inhibits Proliferation and Migration of Glioblastoma Cells through MAPKs Signaling. | (45) |
| Violacein induces p44/42 mitogen-activated protein kinase-mediated solid tumor cell death and inhibits  tumor cell migration. | (61) |
| Xyloketal B Suppresses Glioblastoma Cell Proliferation and Migration in Vitro through Inhibiting TRPM7-Regulated PI3K/Akt and MEK/ERK Signaling Pathways. | (43) |
| JSI-124 Suppresses Invasion and Angiogenesis of Glioblastoma Cells In Vitro. | (62) |
| Coibamide A, a natural lariat depsipeptide, inhibits VEGFA/VEGFR2 expression and suppresses tumor growth in glioblastoma xenografts. | (63) |
| Curzerene suppresses progression of human glioblastoma through inhibition of glutathione S‐transferase A4. | (40) |
| Anti-tumour activity of deer growing antlers and its potential applications in the treatment of malignant gliomas. | (49) |
| Atorvastatin suppresses glioma invasion and migration by reducing microglial MT1-MMP expression. | (11) |
| Simvastatin Inhibits Tumor Growth and Migration by Mediating Caspase-1–Dependent Pyroptosis in Glioblastoma Multiforme. | (64) |
| Apatinib inhibits glioma cell malignancy in patient-derived orthotopic xenograft mouse model by targeting thrombospondin 1/myosin heavy chain 9 axis. | (65) |
| Effect of Blonanserin on the Proliferation and Migration of Glioblastoma Cells. | (50) |
| Fluvoxamine, an anti-depressant, inhibits human glioblastoma invasion by disrupting actin polymerization. | (66) |
| Norepinephrine inhibits migration and invasion of human glioblastoma cell cultures, possibly via MMP-11 inhibition. | (42) |
| Ibuprofen and Diclofenac Restrict Migration and Proliferation of Human Glioma Cells by Distinct Molecular Mechanisms. | (67) |
| Anti-invasive efficacy and survival benefit of the YAP-TEAD inhibitor verteporfin in preclinical glioblastoma models. | (68) |
| Apcin inhibits the growth and invasion of glioblastoma cells and improves glioma sensitivity to temozolomide. | (69) |
| Monensin inhibits glioblastoma angiogenesis via targeting multiple growth factor receptor signaling. | (44) |
| Sulforaphane-cysteine inhibited migration and invasion via enhancing mitophagosome fusion to lysosome in human glioblastoma cells. | (70) |
| Small-molecule agonists of mammalian Diaphanous–related (mDia) formins reveal an effective glioblastoma anti-invasion strategy. | (71) |
| Identification of repaglinide as a therapeutic drug for glioblastoma multiforme. | (51) |

**Appendix Table S3** Quality assessment for in vitro studies (Lewis et al., 2017)

**Remarks:**

1 : Yes

2 : No

3 : Not applicable

Alternative questions:

* : Assessed based on alternative questions (**, ***, ****) mentioned below.

** : Or were control available in the study?

*** : Or were cell lines condition from control and intervention compared?

**** :Or are all results from control and intervention reported?

| **(Author, publication year)** | **Appraisal Questions** (Lewis et al., 2017) | | | | | | **Total scores**  **out of 6** |  |
| --- | --- | --- | --- | --- | --- | --- | --- | --- |
|  | **Have the cells been obtained from a validated repository that guarantees cell verification or have the cells been appropriately independently verified?** | **Have the experiments been performed in sufficient number of times and were appropriate controls included?** | **Were different cell lines from the same cancer type used in the study? **** | **Are culture conditions comparable between different studies?** | **Were cell lines from different cancer types compared? ***** | **Are all results from several cell line experiments reported? ****** |  |  |
| (Saleh et al., 2019) | 1 | 1 | 1 | 1 | 1 | 1 | 6 |  |
| (Rao, Nelson, et al., 2013)* | 1 | 1 | 1 | 1 | 1 | 1 | 6 |  |
| (Bagó et al., 2016) | 1 | 1 | 1 | 1 | 1 | 1 | 6 |  |
| (Ngo et al., 2020)* | 1 | 1 | 1 | 1 | 1 | 1 | 6 |  |
| (Yao et al., 2022)* | 1 | 1 | 1 | 1 | 1 | 1 | 6 |  |
| (C. Wang et al., 2014)* | 1 | 1 | 1 | 1 | 1 | 1 | 6 |  |
| (Rao, DeJesus, et al., 2013)* | 1 | 1 | 1 | 1 | 1 | 1 | 6 |  |
| (Fedorova et al., 2023)* | 1 | 1 | 1 | 1 | 1 | 1 | 6 |  |
| (Jain et al., 2014)* | 1 | 1 | 1 | 1 | 1 | 1 | 6 |  |
| (Lee et al., 2014)* | 1 | 1 | 1 | 1 | 1 | 1 | 6 |  |
| (L.-J. Chen et al., 2021) | 1 | 1 | 1 | 1 | 1 | 1 | 6 |  |
| (W.-L. Chen, Barszczyk, et al., 2015)* | 1 | 1 | 1 | 1 | 1 | 1 | 6 |  |
| (Aroui et al., 2020)* | 1 | 1 | 1 | 1 | 1 | 1 | 6 |  |
|  |  |  |  |  |  |  |  |  |
| (Aroui et al., 2016) | 1 | 1 | 1 | 1 | 1 | 1 | 6 |  |
| (Sun et al., 2022) | 1 | 1 | 1 | 1 | 1 | 1 | 6 |  |
| (Q. Wang et al., 2016) | 1 | 1 | 1 | 1 | 1 | 1 | 6 |  |
| (Guan et al., 2015) | 1 | 1 | 1 | 1 | 1 | 1 | 6 |  |
| (Hei et al., 2019) | 1 | 1 | 1 | 1 | 1 | 1 | 6 |  |
| (Razali et al., 2022)* | 1 | 1 | 1 | 1 | 1 | 1 | 6 |  |
| (Z. Wang et al., 2020) | 1 | 1 | 1 | 1 | 1 | 1 | 6 |  |
| (Rovini et al., 2013)* | 1 | 1 | 1 | 1 | 1 | 1 | 6 |  |
| (Cao et al., 2019) | 1 | 1 | 1 | 1 | 1 | 1 | 6 |  |
| (X. Wang et al., 2017) | 1 | 1 | 1 | 1 | 1 | 1 | 6 |  |
| (Zhang et al., 2021) | 1 | 1 | 1 | 1 | 1 | 1 | 6 |  |
| (Mehta et al., 2015)* | 1 | 1 | 1 | 1 | 1 | 1 | 6 |  |
| (W.-L. Chen, Turlova, et al., 2015)* | 1 | 1 | 1 | 1 | 1 | 1 | 6 |  |
| (Yuan et al., 2015) | 1 | 1 | 1 | 1 | 1 | 1 | 6 |  |
| (Serrill et al., 2016) | 1 | 1 | 1 | 1 | 1 | 1 | 6 |  |
| (Cheng et al., 2022) | 1 | 1 | 1 | 1 | 1 | 1 | 6 |  |
| (Chonco et al., 2021) | 1 | 1 | 1 | 1 | 1 | 1 | 6 |  |
| (Yongjun et al., 2013)* | 1 | 1 | 1 | 1 | 1 | 1 | 6 |  |
| (Yang et al., 2022). | 1 | 1 | 1 | 1 | 1 | 1 | 6 |  |
| (Yao et al., 2021). | 1 | 1 | 1 | 1 | 1 | 1 | 6 |  |
| (Tsuchiya et al., 2023). | 1 | 1 | 1 | 1 | 1 | 1 | 6 |  |
| (Hayashi et al., 2016). | 1 | 1 | 1 | 1 | 1 | 1 | 6 |  |
| (Zhong et al., 2021). | 1 | 1 | 1 | 1 | 1 | 1 | 6 |  |
| (Leidgens et al., 2015). | 1 | 1 | 1 | 1 | 1 | 1 | 6 |  |
| (Barrette et al., 2022). | 1 | 1 | 1 | 1 | 1 | 1 | 6 |  |
| (Ding et al., 2021)* | 1 | 1 | 1 | 1 | 1 | 1 | 6 |  |
| (Wan et al., 2020) | 1 | 1 | 1 | 1 | 1 | 1 | 6 |  |
| (Zhou et al., 2020)* | 1 | 1 | 1 | 1 | 1 | 1 | 6 |  |
| (Xiao et al., 2017)* | 1 | 1 | 1 | 1 | 1 | 1 | 6 |  |

**Appendix Table S4** Quality assessment for in vivo studies (72)

**Remarks:**

6 : Agree strongly

5 : Agree moderately

4 : Agree slightly

3 : Disagree slightly

2 : Disagree moderately

1 : Disagree strongly

CA : I cannot answer

NA : Not applicable

* : Substance refer to the nanofiber materials

** : No experiment bias score 6; experiment bias occurred score 1

*** : No variability score 6; variability available score 1

| **Author, publication year** | **Appraisal Questions** (72) | | | | | | | | | | | | | **Total**  **out of 78** |
| --- | --- | --- | --- | --- | --- | --- | --- | --- | --- | --- | --- | --- | --- | --- |
|  | **Substance** | **Experimental animals** | **Assay** | **Measured effects** | **Tested exposure** | **Laboratory procedures and human factors**** | **Result reporting** | **Result analysis** | **Causal intepretation** | **Results intepretation: epistemological context** | **Results check** | **Results interpretation expert judgement** | **Variability**  ******* |  |
| (Saleh et al., 2019) * | 6 | 6 | 6 | 6 | 6 | 6 | 6 | 6 | 6 | 6 | 6 | 6 | 6 | 78 |
| (Grodecki et al., 2015) * | 6 | 6 | 6 | 6 | 6 | 6 | 6 | 6 | 6 | 6 | 6 | 6 | 6 | 78 |
| (Kievit et al., 2013) * | 6 | 6 | 6 | 6 | 6 | 6 | 6 | 6 | 6 | 6 | 6 | 6 | 6 | 78 |
| (Bagó et al., 2016) * | 6 | 6 | 6 | 6 | 6 | 6 | 6 | 6 | 6 | 6 | 6 | 6 | 6 | 78 |
| (Jain et al., 2014) * | 6 | 6 | 6 | 6 | 6 | 6 | 6 | 6 | 6 | 6 | 6 | 6 | 6 | 78 |
| (Da Silva et al., 2020) | 6 | 6 | 6 | 6 | 6 | 6 | 6 | 6 | 6 | 6 | 6 | 6 | 6 | 78 |
| (Aroui et al., 2020) | 6 | 6 | 6 | 6 | 6 | 6 | 6 | 6 | 6 | 6 | 6 | 6 | 6 | 78 |
| (Sun et al., 2022) | 6 | 6 | 6 | 6 | 6 | 6 | 6 | 6 | 6 | 6 | 6 | 6 | 6 | 78 |
| (Q. Wang et al., 2016) | 6 | 6 | 6 | 6 | 6 | 6 | 6 | 6 | 6 | 6 | 6 | 6 | 6 | 78 |
| (Hei et al., 2019) | 6 | 6 | 6 | 6 | 6 | 6 | 6 | 6 | 6 | 6 | 6 | 6 | 6 | 78 |
| (Z. Wang et al., 2020) | 6 | 6 | 6 | 6 | 6 | 6 | 6 | 6 | 6 | 6 | 6 | 6 | 6 | 78 |
| (Serrill et al., 2016) | 6 | 6 | 6 | 6 | 6 | 6 | 6 | 6 | 6 | 6 | 6 | 6 | 6 | 78 |
| (Cheng et al., 2022) | 6 | 6 | 6 | 6 | 6 | 6 | 6 | 6 | 6 | 6 | 6 | 6 | 6 | 78 |
| (Yao et al., 2021). | 6 | 6 | 6 | 6 | 6 | 6 | 6 | 6 | 6 | 6 | 6 | 6 | 6 | 78 |
| (Hayashi et al., 2016). | 6 | 6 | 6 | 6 | 6 | 6 | 6 | 6 | 6 | 6 | 6 | 6 | 6 | 78 |
| (Barrette et al., 2022). | 6 | 6 | 6 | 6 | 6 | 6 | 6 | 6 | 6 | 6 | 6 | 6 | 6 | 78 |
| (Wan et al., 2020) | 6 | 6 | 6 | 6 | 6 | 6 | 6 | 6 | 6 | 6 | 6 | 6 | 6 | 78 |
| (Arden et al., 2015). | 6 | 6 | 6 | 6 | 6 | 6 | 6 | 6 | 6 | 6 | 6 | 6 | 6 | 78 |
| (Xiao et al., 2017). | 6 | 6 | 6 | 6 | 6 | 6 | 6 | 6 | 6 | 6 | 6 | 6 | 6 | 78 |

**Appendix Table S5** Biomimetic techniques and material used as nanofibers

| **Biomimetic techniques** | **Materials for biomimetic techniques** | **Type of GBM models** | **Type of cells** | **Development stage** | **(Author, publication year)** |
| --- | --- | --- | --- | --- | --- |
| Nanofibers to mimic white matter tracts. | Polyacrylonitrile (PAN)-derived nanofiber (NF) | neurosphere | Gli4 and GliT | in vitro and in vivo | (Saleh et al, 2019) |
|  | Electrospun poly(caprolactone) (PCL) nanofiber scaffolds |  | Patient-derived OSU-2 Cell Culture | in vivo | (Grodecki et al., 2015) |
|  | Aligned chitosan-polycaprolactone polyblend nanofibers (Chitosan-PCL polyblend nanofibers) |  | Patient-derived OSU-2 cell culture | in vivo | (Kievit et al., 2013) |
|  | Core-shell electrospun   - Core: gelatin, poly(ether sulfone), poly(dimethylsiloxane)) - Shell: poly(ε-caprolactone) (PCL) material to conserve surface chemistry |  | Patient-derived OSU-2 cell culture | in vitro | (Rao et al., 2013b) |
|  | Poly(L-lactic acid) Biocompatible electrospun nanofibrous scaffolds (bENS) or  PLA bENS |  | U87, LN229 and U251 human glioma cell | in vitro and in vivo | (Bagó et al., 2016) |
| **Total studies: 5*** | | | | | |

**Remarks:**

*: There were 5 studies using nanofibers as biomimetic techniques

**Appendix Table S6** Biomimetic techniques and material used as hydrogels and other extracellular matrix

| **Biomimetic techniques** | **Materials for biomimetic techniques** | **Type of GBM models** | **Type of cells** | **Development stage** | **(Author, publication year)** |
| --- | --- | --- | --- | --- | --- |
| Hydrogels to mimic the brain ECM. | Hydrogel from gelatin methacrylamide (GelMA) | neurosphere | U87-MG | in vitro | (Ngo et al., 2020) |
|  | Collagen hydrogel |  | Primary human glioma cells (HCM-BROD-0002-C71, ATCC, Manassas, VA, USA) | in vitro | (Yao et al., 2022) |
|  | PEG-based hydrogels |  | U87 glioma cells | in vitro | (Wang et al., 2014) |
|  | Collagen hyaluronan composite hydrogels (from collagen and thiolated HA) |  | Patient Tumor Derived OSU-2 Cell Culture | In vitro | (Rao et al., 2013a) |
| Other technique to mimic brain ECM. | GLICO model using poly-HEMA-treated nonadherent |  | U87-GFP | In vitro | (Fedorova et al., 2023) |
| **Total studies with hydrogels: 4***  **Total other study: 1*** | | | | | |

**Remarks:**

*: There were 4 studies using hydrogels and 1 other study using GLICO culture as biomimetic technique

**Appendix Table S7** Biomimetic techniques and material used in biomimetic techniques utilizing the combination of biomimetic techniques

| **Biomimetic techniques** | **Materials for biomimetic techniques** | **Type of GBM models** | **Type of GBM cells** | **Development stage** | **(Author, publication year)** |
| --- | --- | --- | --- | --- | --- |
| Combination of nanofibers and hydrogels | - PCL-aligned nanofibers, - Collagen hydrogel or a cyclopamine-conjugated collagen hydrogel. | neurosphere | U87MG-eGFP | In vitro and in vivo | (Jain et al., 2014) |
|  | - Hydrogel with acrylation of hyaluronic acid and microfluidic chip, - Electrospun nanofiber membrane from polyurethane (PU) |  | glioma cell line A-172 (American Type Culture Collection [ATCC] | In vitro | (Lee et al., 2014) |
| **Total studies: 2*** | | | | | |

**Remarks:**

*: There were 2 studies using a combination of nanofibers and hydrogels as biomimetic techniques

**Appendix Table S8** Source of natural compounds as potential anti-migratory drugs for GBM treatment

| **Group of natural substances** | **Natural compound name** | **Source of compound** | **Previously identified activities** | **Migration and invasion assays** | **Other supporting assays** | **Type of GBM cells used** | **Development stage** | **Molecular mechanism studied related to GBM migration and invasion** | **(Author, publication year)** |
| --- | --- | --- | --- | --- | --- | --- | --- | --- | --- |
| Flavonoid | Flavonoid rutin and aglycone quercetine* | Brazilian plant *Dimorphandra mollis Bent* | Gastroprotective, hepatoprotective, anti-diabetic effects, anti-inflammatory, anti-glycation activities, and reduction of anxiety. | Wound healing and transwell assays | - Cell proliferation assay using Bromodeoxyuridine - Molecular mechanism assay (ELISA assay for cytokines) | U251 and TG1 glioblastoma cells | in vitro and in vivo | - Upregulating the expression of IL-1β, IL-18, CX3CL1, PTGS2 and NOS2 - Downregulating the expression of IL-6, TGFβ, arginase, HDGF, and IGF (Da Silva et al., 2020) | (Da Silva et al., 2020) |
|  | Luteolin and apigenin* | *Wedelia chinensis* | Anticancer properties effective against prostate, lung, breast, glioblastoma, colon, and pancreatic cancer cells | Transwell migration assay | - Cell apoptosis assay using immunoblotting and - Autophagy assay using monitoring Autophagy-Immunofluorescence Staining | Human malignant glioma cell lines, GBM8401 and U-87MG,  and human brain astrocyte (HBA) cells | in vitro | Not yet studied | (Chen et al., 2021) |
|  | Carvacrol | Naturally synthesized | Antimicrobial, antibacterial, antiviral, antifungal, and antiprotozoal based on previous studies | Wound healing and transwell assays | - Cell proliferation assay using MTT assay - Cell apoptosis assay - Molecular mechanism assay | U87 | in vitro | Inhibiting TRPM7 (Chen et al., 2015a) | (Chen et al., 2015a) |
|  | Naringin  (flavanones)** | Citrus fruits | Antioxidant and anti-cancer-related properties | Transwell migration assay | In vitro:   - Cell proliferation assay using MTT assay | Human malignant glioma cell line, U87 | in vitro and in vivo | - Inducing by VEGF in a dose-dependent manner which caused inhibition of the activation of VEGFR2 after VEGF stimulation, Inhibition of p-AKT and p-ERK caused inhibition of the phosphorylation of p-AKT and p-ERK (Aroui et al., 2020) - Inhibiting the phosphorylation of JNK1/2, ERK1/2 and p38 pathways (Aroui et al., 2016) | (Aroui et al., 2020) |
|  |  |  |  |  | In vivo using a xenograft mouse model   - Molecular mechanism assays in vivo |  |  |  |  |
|  |  |  |  | Wound healing assay  Boyden chamber | - Cell viability and cell cycle distribution assay - Molecular mechanism assay | U87 and U373 | in vitro |  | (Aroui et al., 2016) |
| Alkaloid | Berberine | Not mentioned | Traditional Chinese medicines for bacterial diarrhea | Wound healing assay | In vitro:   - Proliferation cell assay - Molecular mechanism assay | Human glioma cells U87 MG, U251, U118 MG, A172, T98G, LN18, LN229, and human umbilical vein endothelial cell (HUVEC) | in vitro and in vivo | Inhibiting the expression of COL11A1 (Sun et al., 2022) | (Sun et al., 2022) |
|  |  |  |  |  | In vivo using mouse xenografts: Molecular mechanism assay in vivo |  |  |  |  |
|  | Kukoamine A | Cortex *Lycii radicis* | Traditional Chinese herb and has biological effects for antihypertensive, antioxidant, anti-inflammatory, soybean lipoxygenase inhibition | Wound healing and transwell chamber assay | In vitro:   - Proliferation assay using MTT assay - Cell apoptosis assay using AO/EB staining apoptotic cells - Cell cycle analysis | U251and WJ1 | in vitro and in vivo | Upregulating E-cadherin expression and downregulating C/EBPβ, N-cadherin, vimentin, twist, and snail+ slug expressions (Wang et al., 2016) | (Wang et al., 2016) |
|  |  |  |  |  | In vivo: GBM growth in vivo assay |  |  |  |  |
| Glycosides | Ginsenoside Rh2  (steroid glycosides) | Red ginseng | Therapeutic effects on inflammation and a number of cancers | Scratch wound healing assay and transwell cell migration assay | Molecular mechanism assay | The human glioma cell line U251,  Patient-derived cell | in vitro | - Inhibiting PI3k/Akt signaling - Downregulating MMP, MMP2, MMP9, and MMP13 | (Guan et al., 2015) |
|  | Verbascoside  (phenylethanoid glycoside) | Lemon verbena | Anti-inflammation, antioxidant, and immunomodulation | Transwell chamber | In vitro:  Proliferation cell assay | The human GBM cell lines U87, U251 and U87-luciferase | in vitro and in vivo | Reducing c-Met through the proteasome ubiquitination pathway (Hei et al., 2019) | (Hei et al., 2019) |
|  |  |  |  |  | In vivo: U87 xenograft mouse model with VB  Molecular mechanism assay in vivo |  |  |  |  |
| Curcuminoids | Curcumin analogues (FLDP-5 and FLDP-8)*** | Turmeric | Anti-inflammatory, cytotoxicity, and apoptosis induction on several cancer cell lines | Wound scratch assay | - Proliferation cell assay using MTT assay - Cell apoptosis assay - Cell cycle analysis | LN-18 human GBM cells. | in vitro | - Inducing cycle termination in U-18 cells to inhibit invasion of GBM cells. - Inducing oxidative stress through the generation of ROS superoxide anion, which plays an important role in inducing cell apoptosis (Razali et al., 2022) | (Razali et al., 2022) |
|  | Curcumin (1,7-bis(4-hydroxy-3-methoxyphenyl)-1,6-heptadiene3,5-dione)*** | Turmeric of Curcuma | Adjunctive therapy in some inflammatory and neurodegenerative diseases | Scratch wound healing assays | In vitro:   - Cell proliferation assay - Cell apoptosis assay using The Annexin V-fluorescein isothiocyanate (FITC)-propidium iodide (PI) apoptosis Kit | U251and U87 | in vitro and in vivo | - Increasing the expression of PTEN and p53 (Wang et al., 2020) | (Wang et al., 2020) |
|  |  |  |  |  | In vivo:  Antitumor activity assay |  |  |  |  |
| Vinca alkaloids | Vinflunine | Not mentioned | Approved indication by EMA as treatment of adults with advanced or metastatic urothelial cancers | Wounding-healing assay, transwells | - Cell proliferation assay using MTT assay - Molecular mechanism assay | Human glioblastoma (U87MG) | in vitro | Inhibiting EB1 accumulation (Rovini et al., 2013) | (Rovini et al., 2013) |
| Triterpenoid saponin | Tubeimoside-1 | Rhizoma bolbostemmae | Traditional Chinese medicine as an anti-cancer function in the treatment of esophagus and gastric cancer | Wound-healing assay and Transwells assay | In vitro:   - Cell proliferation assay BrdU Staining - Cell cycle analysis - Molecular mechanism assay | U87 and LN229 cells | in vitro and in vivo | Enhancing the level of ubiquitination of MET (Cao et al., 2019) | (Cao et al., 2019) |
|  |  |  |  |  | In vivo using tumor xenograft |  |  |  |  |
| Sesquiterpene lactone | Alantolactone | *Inula helenium* | antibacterial, antifungal, anti-inflammatory, and hepatoprotective activities | Wound healing assays and transwell assays | In vitro:   - Cell apoptosis assay - BBB penetration assay (Detection of ATL through the BBB) - Cell cycle arrest assay - Molecular mechanism assay | U87 and U251 | in vitro and in vivo | Inhibiting COX-2 signaling in human GBM cells | (Wang et al., 2017) |
|  |  |  |  |  | In vivo: tumor cell–inoculated mice  Tumor growth inhibition in vivo |  |  |  |  |
| Phytocannabinoids | Cannabisin D | *Sinomenium Acutum* | Traditional Chinese Medicine as treatment for inflammatory and rheumatic diseases | Transwell migration assay | - Cell proliferation assay - Cell viability assay - Cell apoptosis assay - Cell cycle analysis | The human glioblastoma cell lines U-251 and U-87 | in vitro | Inhibiting MAPKs Signaling (Zhang et al., 2021) | (Zhang et al., 2021) |
| Hydroxyindoles | Violacein | *Chromobacterium violaceum* | a purple pigment | Boyden chamber assays | - Cell proliferation assay using crystal violet - Molecular mechanism assay | U87 (glioblastoma) | in vitro | Inducing p44/42 mitogen-activated protein kinase (Mehta et al., 2015) | (Mehta et al., 2015) |
| Pentacyclic fungal, dimethyl analogs | Xyloketal B | Mangrove fungus *Xylaria sp* | Not mentioned | Wound healing assay | - Cell proliferation assay - Cell viability assay - Molecular mechanism assay | U251 cells. | in vitro | Suppressing TRPM7 and modulating PI3K/Akt and MEK/ERK signaling pathways (Chen et al., 2015b) | (Chen et al., 2015b) |
| Cucurbitacin | JSI-124 (cucurbitacin I) | Not mentioned | Inhibitor of STAT3 inhibitor and induce the apoptosis of human tumor cell lines | Transwell migration | In vitro:   - Cell apoptosis assay - Cell viability assay - Molecular mechanism assay | U251 and U87MG | in vitro and in vivo | Inhibiting VEGF secretion (Yuan et al., 2015) | (Yuan et al., 2015) |
|  |  |  |  |  | In vivo using tumor xenograft model: Molecular mechanism assay |  |  |  |  |
| Cyclodepsipeptide | Coibamide A | Marine cyanobacteria | Inhibitor growth of cancer cell lines | Transwell migration assays | In vitro:   - Cell proliferation assay - Molecular mechanism assay | U87-MG glioblastoma  Human SF-295 glioblastoma cells. | in vitro and in vivo | Inhibiting VEGFA/VEGFR2 expression and suppressing tumor growth in glioblastoma xenografts (Serrill et al., 2016) | (Serrill et al., 2016) |
|  |  |  |  |  | In vivo using U87-MG GBM xenograft model in mice:  Anti-tumor assay |  |  |  |  |
| Terpenoid | Curzerene | *Curcuma longa* | Traditional Chinese medicine as anticancer agents | Wound healing assay and transwell migration assay | In vitro:   - Cell proliferation assay - Cell apoptosis assay - Molecular mechanism assay | U251 and U87 | in vitro and in vivo | Inhibiting the activation of the mTOR pathway and the expression of MMP9 (Cheng et al., 2022) | (Cheng et al., 2022) |
|  |  |  |  |  | In vivo using tumor xenograft growth in nude mice:  Observation of the survival time of tumor-bearing mice |  |  |  |  |
| Nucleosides | Deer antler velvet (DAV) extract | Red deer (*Cervus elaphus*), sika deer (*Cervus nippon*), white-tailed deer (*Odocoileus virginianus*), and elk (*Cervus canadensis*) | Anti-cancer | Scratch assay. | - Cell proliferation assay - Cell apoptosis assay - Cell viability assay - Cell cycle analysis | T98G and A172 | in vitro | Not yet studied | (Chonco et al., 2021) |
| **Total studies: 21**  **Total compounds: 20 natural compounds** | | | | | | | | | |

**Remarks:**

* : count as one compound because they came from the same source and were tested together

** : count as one compound which had two different studies

*** : count as two compounds because one study used curcumin and the other one used analogs of curcuminoid

**Appendix Table S9** Potential anti-migratory compound for GBM treatment from small molecules and biologic

| **Type of compound** | **Compound name** | **Repurposed medicines or not** | **Previous approved Indication** | **Migration assay** | **Other supporting assays** | **Type of GBM cells** | **Development Stage** | **Molecular mechanism**  **related to GBM migration and invasion** | **(Author, year)** |
| --- | --- | --- | --- | --- | --- | --- | --- | --- | --- |
| Small molecules | Atorvastatin | Yes | Dyslipidaemia treatment acting as inhibitors of 3-hydroxy-3-methylglutaryl-coenzyme A (HMG-CoA) reductase | Transwell assay | - Cell viability assay - Molecular mechanism assay | A human U87 human primary glioblastoma cell line | In vitro | - Reducing microglial MT1-MMP expression (Yongjun et al., 2013) - Inhibiting the phosphorylation status of JNK in U87 GBM cells. (Yongjun et al., 2013) | (Yongjun et al., 2013). |
|  | Simvastatin | Yes | Dyslipidaemia treatment acting as inhibitors of 3-hydroxy-3-methylglutaryl-coenzyme A (HMG-CoA) reductase | Wound-Healing assay, Transwell Assay | - Cell viability assay - Cell cycle analysis - Molecular mechanism assay | U87 and U251 human glioma cell lines w | In vitro | Inhibiting Caspase-1 (Yang et al., 2022) | (Yang et al., 2022). |
|  | Apatinib | Yes | Non-small cell lung cancer (NSCLC) treatment | Scratch test and transwell assay | In vitro:   - Cell proliferation assay - Molecular mechanism assay | Cell culture Two primary human GBM cell lines (N14042 and N14069) were obtained from two GBM patients and the commercial U87 cell line. | In vivo and in vitro | Targeting thrombospondin 1/myosin heavy chain 9 axis (Yao et al., 2021). | (Yao et al., 2021). |
|  |  |  |  |  | In vivo using patient-derived orthotopic xenograft (PDOX) glioma mouse model:  Progression of GBM cells in vivo assay |  |  |  |  |
|  | Blonanserin | Yes | Treatment of schizophrenia. | Scratch assay | - Cell proliferation assay - Cell viability assay - Cell growth inhibitory assay | Human glioblastoma cell lines (U251 and T98G cells) | In vitro | Not yet studied | (Tsuchiya et al., 2023). |
|  | Fluvoxamine | Yes | Antidepressant | Wound-healing assay  Matrigel invasion assay | In vitro:   - Cell proliferation assay using WST-1 assay - Actin polymerization assay - In vitro actin assembly assay. - Molecular mechanism assay | Human GBM cell lines U87-MG, U251-MG, and A172 | In vitro and in vivo | Inhibiting FAK and Akt/mTOR signaling. (Hayashi et al., 2016) | (Hayashi et al., 2016). |
|  |  |  |  |  | In vivo using GBM xenograft:  Cell proliferation assay |  |  |  |  |
|  | Norepinephrine | Yes | Blood pressure control in certain acute hypotensive states | Wound Healing Assay  Matrigel or transwell invasion assay | Molecular mechanism assay | Malignant brain tumor cell lines U251 and U87 | In vitro | Downregulating MMP-11 expression (Zhong et al., 2021) | (Zhong et al., 2021). |
|  | Ibuprofen and Diclofenac* | Yes | Inflammatory diseases, rheumatoid disorders, and mild to moderate pain | Spheroid migration assays | - Cell proliferation assay - Cell cycle analysis - Molecular mechanism assay | Human high-grade glioma cell lines U87MG and A172 | In vitro | Decreasing STAT-3 phosphorylation and decreasing c-myc expression (Leidgens et al., 2015) | (Leidgens et al., 2015)**.** |
|  | Verteporfin (VP) | Yes | Macular degeneration treatment as an inhibitor of YAP-TEAD19 and TAZTEAD | Spheroid dispersion/migration assay and transwell invasion assays | In vitro:   - Cell proliferation assay - Cell viability assay - Molecular mechanism assay | 8 patient-derived lines at low passage (<30) (G-13063, G-12746, G-13181, G-16302, G-13514, G-17969, G-11849, G-11849R) and U87 GBM cell line | In vitro and in vivo | Inhibiting YAP, TEAD1, and TEAD1-target expression in PDX tumors (Barrette et al., 2022) | (Barrette et al., 2022). |
|  |  |  |  |  | In vivo using Patient-derived orthotopic xenograft (PDX):  Cell proliferation assay |  |  |  |  |
|  | Apcin | N/A | N/A | Wound healing assay | - Cell apoptosis assay - Cell viability assay - Molecular mechanism assay | U251MG | In vitro | Increasing Bim expression in GBM cells after being treated with apcin (Ding et al., 2021) | (Ding et al., 2021). |
|  | Monensin | N/A | N/A | Transwell assay | In vitro   - Cell proliferation assay - Cell apoptosis assay - Cell adhesion assay - Cell growth and survival - Molecular mechanism assay | Human glioblastoma cell lines, U87, U251MG, T98G, A172, U251 N and U373 and HCC287 | in vitro and in vivo | Inhibiting VEGR and EGFR signaling pathways in endothelial cells | (Wan et al., 2020) |
|  |  |  |  |  | In vivo:  Inhibition angiogenesis and growth of GBM in vivo |  |  |  |  |
|  | Sulforaphane-cysteine (SFN-Cys) | N/A | N/A | Wound scratch healing assay,  transwell chamber assay | - Autophagy and mitophagy assay - Molecular mechanism assay | Human GBM U87MG | In vitro | Inhibiting Claudin-5 and S100A4 (Zhou et al., 2020) | (Zhou et al., 2020). |
|  | Small-molecule agonists of mammalian Diaphanous–related (mDia) or mDia agonism | N/A | N/A | Scratch assay, fluoroblock transwell chamber assay | Ex vivo: Spheroid invasion in an ex vivo brain-slice invasion model | U87, U343, U118, T98G, SNF19, SF295, LN229, and A172 | ex vivo | Enhancing F-actin dynamics (Arden et al., 2015) | (Arden et al., 2015). |
| Biologics | Repaglinide | Yes | Oral insulin secretagogue for patients with type 2 diabetes mellitus | Scratch-wound culture model and cell migration assay | In vitro:   - Cell proliferation assay using MTT assay - Cell viability assay | Human GBM cells (LN229). | In vitro and in vivo | Not yet studied, however the discussion mentioned that the probability of repaglinide could inhibit GBM cell migration via downregulating Blc-1, Beclin-1, and PD-L1. | (Xiao et al., 2017). |
|  |  |  |  |  | In vivo using orthotopic GBM model:   - Observation median survival time - Molecular mechanism |  |  |  |  |
| **Total studies: 13**  **Total compounds: 13 (12 small molecules and 1 biologic)** | | | | | | | | | |

**Remarks:**

* : count as a single compound combination because they were tested together in the same study
